# Palliative, palliative or palliative?

**DOI:** 10.1186/s13054-021-03633-2

**Published:** 2021-06-10

**Authors:** René Robert, Michel Goldberg

**Affiliations:** 1 Service de Médecine Intensive Réanimation, University of Poitiers, CHU Poitiers France, CIC Inserm 1402 CHU, 86000 Poitiers, France; 2 University of La Rochelle France, CNRS, 7266 LIENSs, La Rochelle, France

## Introduction

Palliative care has emerged as a crucial component in the medical course of many patients, particularly in cancer, neurodegenerative diseases, advanced heart, respiratory or liver failure [1, 2]. However, the single word “palliative” is uniformly used, whatever the stages of disease evolution, and the specific therapeutic avenues that may remain possible. In case of acute altered organ function, the palliative patient "label" can lead to therapeutic withholding or withdrawal of life support strategies, at times even entailing refusal of ICU admission. It would make sense to use more nuanced and discriminating terminologies to clarify different clinical situations.

## The same word covers three different stages of the disease

The term “palliative” reflects a clinical situation without prospect of complete recovery from the illness. It is defined as treatment that alleviates the symptoms of a disease without acting on its causes. Palliative care integrates the management of physical and psychological pain and other symptoms that are bothersome to the patient. In several clinical situations, it begins early in the patient's life when his prognosis is still good; this initial stage implies prolonged life expectancy and normal or sub-normal quality of life (QOL). At a latter stage, the end of life is approaching. Physical condition is altered, activities are reduced and the patient may need partial assistance with some daily living activities. Mental capacities are generally close to normal. QOL is limited but may still be considered acceptable based on the patient's self-evaluation. At this stage of the disease, curative care is reaching the end of its intended effect and the palliative care has increased [3]. Lastly, when the end of life is near, physical capacities are extremely poor, QOL is deeply altered and the patient needs assistance with all daily living activities. Overall distinction between these three stages is associated with prognosis of diseases, co-morbidities, QOL evaluation. However, the boundary between the different stages is not clear, and healthcare teams need to decipher the grey areas between them.

Taking the three stages of the patient's course outlined above, we can schematically define an appropriate management strategy. During the first phase, the patient should be considered as unrestricted and benefit from unlimited treatment. If necessary, he or she can be admitted to an ICU with full code management. When the patient is approaching the end of life, on the other hand, treatment may be withheld. Given the complications, the probability of dying soon is high, but there remains hope in survival of the patient with the goal of restoring a satisfactory QOL in the short, medium and occasionally long term. As an example, patients admitted to ICU for respiratory distress with a do not intubate order may benefit from non-invasive methods of oxygenation or ventilation [4]. Finally, at the end of life stage, specific treatments are withdrawn, and the expected outcome is death of the patient. The priority is to ensure the best possible quality of dying in accordance with the patient's wishes, integrating support for family.

## More discriminating terminologies to clarify differing situations

Different terms have been used to more closely correspond to the clinical condition of the palliative care patient: "comfort", "supportive", "best supportive care" or “hospice care” [5–7]. Whatever the term, confusion in terminology persists and no semantic consensus has been reached [8]. Heterogeneity is also found to characterize the end of life: “end-of-life”, “terminal illness”, “actively dying” and corresponding ways to treat the patient: "care of the dying", "terminal care", “transition of care”, "quality-of-life care" [6]. Moreover, the implied duration of remaining life for end-of-life patients can range from a few days to 6 months, highlighting the confusion surrounding the ambiguities regarding the nature and intensity of the care to be provided.

Since up until now, no single word has been satisfactory, it is crucial to identify one or more words that can consensually clarify the patient's care pathways. The terminology should apply regardless of the pathology concerned.

## Distinction between the characterization of care and stage of the disease

Care refers to a therapeutic objective that can be preventive or directly contribute to clinical improvement of the patient. Stage refers to the evolution of the disease according to various kinetics. The term palliative "care" can be applied to the patient without prejudging disease prognosis whereas palliative "stage" corresponds to a pejorative prognosis for the patient.

## Lexical approach

To overcome historical ambiguities and to improve description of a patient’s course we propose new terms to refine the semiology of clinical situations. Firstly, we suggest the new word "pallitative" to characterize palliative care focused on end-of-life support. This neologism would apply to patients whose death is expected within a few days or a few weeks without any curative treatment or vital support treatment, and for whom comfort care at the end of life is a priority. The term “pallitative” can be considered as a lexical amalgam based on a common thematic sound; with this neologism, the general field of palliative care remains intact.

Secondly, we suggest the term “meliorative”. Seldom used, it is opposed to “pejorative”. It comes from the Latin *meliorare*, which means to improve, presenting the designated idea or object in a favorable light. It could be applied to the need to maintain the best possible QOL in situations where that is the objective. There is no longer any prospect of recovery or substantial expected improvement in the patient's condition. Patients benefit primarily from symptomatic treatment and care aimed at improving their immediate well-being.

In all clinical situations, the palliative care terminology remains intact, maintaining a global vision of the management of the seriously ill patient.

In summary, enriching the word "palliative" with semantic additions—including the word "meliorative" and the neologism "pallitative"- is proposed as a way of more precisely characterizing the nature of palliative care for the seriously ill (Table 1). This approach would be concordant with the physician orders for life-sustaining treatment (POLST) orders and facilitate preference concordance [9]. Such new proposals need to be discussed, validated and appropriated by the caregivers involved.

**Table 1:** New proposals to characterize palliative management

	Temporality	General condition physical abilities	Goals of Care	Admission in ICU
Stage 1 Curative	Extended life expectancy with good expected quality of life	Preserved or can be standardized	Curative unlimited	Yes
Stage 2 Meliorative	Estimated end of life less than 6 months and altered quality of life	Altered	Sustaining quality of life	No, or yes by whithholding life support treatment
Stage 3 Pallitative	Entering the end of life; death expected within a few days to a few weeks	Deeply altered	End-of-life support	No

## Data Availability

Not applicable.
